# Early signs of architectural and biomechanical failure in isolated myofibers and immortalized myoblasts from desmin-mutant knock-in mice

**DOI:** 10.1038/s41598-017-01485-x

**Published:** 2017-05-03

**Authors:** Stefanie Diermeier, Julian Iberl, Kristina Vetter, Michael Haug, Charlotte Pollmann, Barbara Reischl, Andreas Buttgereit, Sebastian Schürmann, Marina Spörrer, Wolfgang H. Goldmann, Ben Fabry, Fatiha Elhamine, Robert Stehle, Gabriele Pfitzer, Lilli Winter, Christoph S. Clemen, Harald Herrmann, Rolf Schröder, Oliver Friedrich

**Affiliations:** 1 Institute of Medical Biotechnology, Friedrich-Alexander Universität Erlangen-Nürnberg, Paul-Gordan-Str.3, 91052 Erlangen, Germany; 2 SAOT, Erlangen Graduate School in Advanced Optical Technologies, Friedrich-Alexander-Universität Erlangen-Nürnberg, Erlangen, Germany; 3 Department of Physics, Biophysics Group, Friedrich-Alexander-Universität Erlangen-Nürnberg, Erlangen, Germany; 4 Institute for Vegetative Physiology, University of Cologne, Cologne, Germany; 5 Institute of Neuropathology, University Hospital Erlangen, Erlangen, Germany; 6 Institute of Biochemistry I, Medical Faculty, University of Cologne, Cologne, Germany; 7 Department of Neurology, Heimer Institute for Muscle Research, University Hospital Bergmannsheil, Ruhr-University Bochum, Bochum, Germany; 8 Muscle Research Center Erlangen (MURCE), Friedrich-Alexander-Universität Erlangen-Nürnberg, Erlangen, Germany

## Abstract

In striated muscle, desmin intermediate filaments interlink the contractile myofibrillar apparatus with mitochondria, nuclei, and the sarcolemma. The desmin network’s pivotal role in myocytes is evident since mutations in the human desmin gene cause severe myopathies and cardiomyopathies. Here, we investigated skeletal muscle pathology in myofibers and myofibrils isolated from young hetero- and homozygous R349P desmin knock-in mice, which carry the orthologue of the most frequent human desmin missense mutation R350P. We demonstrate that mutant desmin alters myofibrillar cytoarchitecture, markedly disrupts the lateral sarcomere lattice and distorts myofibrillar angular axial orientation. Biomechanical assessment revealed a high predisposition to stretch-induced damage in fiber bundles of R349P mice. Notably, Ca^2^^+^-sensitivity and passive myofibrillar tension were decreased in heterozygous fiber bundles, but increased in homozygous fiber bundles compared to wildtype mice. In a parallel approach, we generated and subsequently subjected immortalized heterozygous R349P desmin knock-in myoblasts to magnetic tweezer experiments that revealed a significantly increased sarcolemmal lateral stiffness. Our data suggest that mutated desmin already markedly impedes myocyte structure and function at pre-symptomatic stages of myofibrillar myopathies.

## Introduction

The intermediate filament (IF) protein desmin is a key component of the three-dimensional, filamentous extra-sarcomeric cytoskeleton, which interlinks neighboring myofibrils at the level of Z-discs and connects the whole myofibrillar apparatus with costameres, intercalated discs, myotendinous and neuromuscular junctions as well as nuclei and mitochondria in striated muscle cells^1–4^. Hence, the desmin network provides prime architectural anchorage for structural and functional lateral alignment of myofibrils. In addition to a putative role in mechanical signaling, desmin filaments may exert important functions in the adaptation of striated muscle fibers to mechanical stress generated by their contractile performance and during passive stretch^5, 6^. Desmin’s essential role is highlighted by the fact that human desmin gene (DES) mutations (chromosome 2q35) cause myopathies and cardiomyopathies^7, 8^. Desminopathies are classical protagonists of a clinically and genetically diverse group of myofibrillar myopathies; which are morphologically characterized by desmin-positive protein aggregates and myofibrillar degeneration^9–11^. Clinically, desminopathies are highly variable: onsets range from the first to the eighth life decade and disease manifestations comprise pure myopathy, cardiomyopathy, or both^12, 13^. The majority of desminopathies is due to heterozygous mutations accounting for autosomal-dominant cases while more rare recessive cases are further subdivided into those with maintained mutant desmin expression^7, 14–17^ and, even more rarely, others with complete lack of desmin^18–20^. Since human muscle tissue from preclinical disease stages is usually not available, patient-mimicking animal models are needed to study the pathogenesis of desminopathies. We recently engineered and characterized hetero- and homozygous R349P desmin knock-in (Des^R349P^) mice harboring the orthologue of the most frequently occurring human missense mutation R350P and display age-dependent skeletal muscle weakness and cardiomyopathy^21^.

Here, we used this model for quantitative *Second Harmonic Generation* (SHG) microscopy with ~µm³ resolution and label-free imaging of sarcomeric myosin in 3D^22^. Our combined morphological-biomechanical investigation unveils early, pre-clinical desminopathy disease patterns where mutant desmin disrupts the extra-sarcomeric intermediate filament network, causing aberrant myofibrillar alignment and orientation. These changes provide a structural explanation for compromised force production in symptomatic disease stages in addition to significantly increased stiffness and stretch-induced vulnerability.

## Results

### The experimental strategy

**Figure 1:**
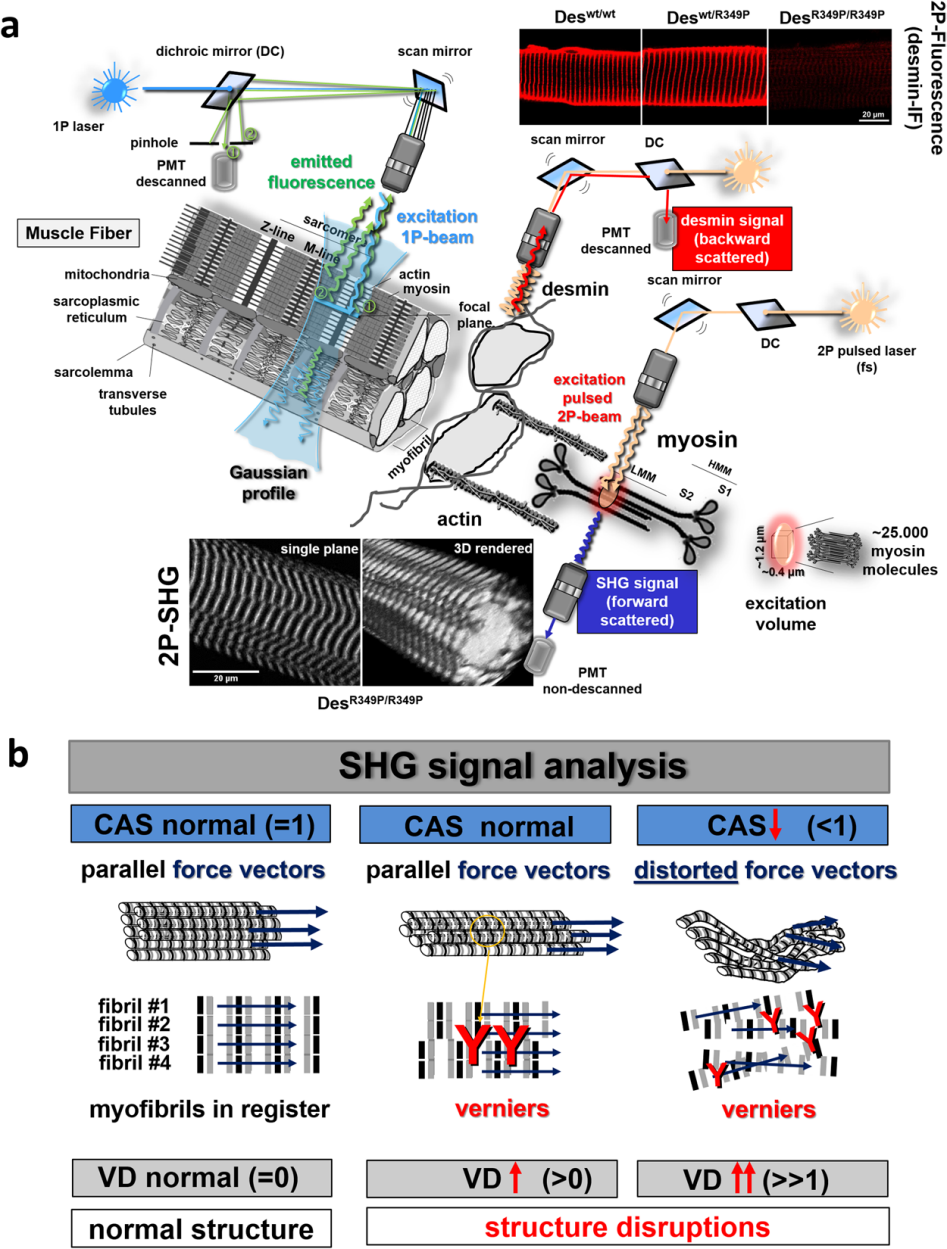
Two-photon (2P) microscopy by SHG and desmin 2P immunofluorescence in single fibers from Des^R349P^ mice. (**a**) Optical beam path in 2P imaging of intrinsic myosin-derived SHG signals or 2P-desmin immunofluorescence confined to a tiny excitation volume of about 1 µm^3^ within the tissue (left part) in comparison to conventional 1P-confocal imaging. Although off-plane emission signals (marked as ②) are blocked from detection by the pinhole, confocal imaging suffers from Gaussian beam profiles during excitation of the whole z-depth during point-scanning. This gives rise to photo-bleaching and photo-damage while obtaining XYZ stacks in thick samples, like muscle fibers. Also, 1P-imaging requires external labels at all times. In contrast, non-linear 2P-imaging takes advantage of signals from selected intrinsic proteins, such as myosin II in muscle. This allows to minimal-invasively obtain detailed 3D views of sarcomere ultrastructure within XYZ stacks through single fibers. This 3D visualization of myofibrillar geometry is not possible from trans-illumination imaging or in histology sections. Examples from single plane SHG images as well as a 3D rendered XYZ volume from a SOL single fiber of a hom Des^R349P^ mouse are shown (left lower panel) as well as desmin 2P-signals from wt mice. (**b**) Image processing algorithms applied to obtain SHG 3D volumes allow a quantitative morphometry approach using *cosine angle sums* (CAS) and *vernier densities* (VD) as morphological parameters to describe disturbances in muscle architecture as a morphological correlate for muscle weakness.

Our previously generated knock-in mouse strain allowed us to derive myofibers that are hetero- and homozygous for R349P mutant desmin to investigate their structural properties by SHG microscopy (Fig. 1). We performed a detailed quantitation of the myofibrillar cytoarchitecture by extracting two morphometric parameters from the images^23, 24^: (i) the *cosine angle sum* (CAS), a summed projection of the angular orientations from all myofibrils and (ii), the so-called *vernier density* (VD). CAS serves as a measure for the coherency and structural integrity of the contractile apparatus and allows an estimate for the projected force generation of single muscle fibers (Fig. 1). The VD quantitates out-of-register Y-shaped deviations from the regular parallel striation pattern of adjacent myofibrils that can be automatically detected, counted, and normalized to muscle fiber volume (Fig. 1b). Furthermore, we performed comprehensive measurements of active and passive biomechanical properties in muscle fiber bundles, thin myofibrillar bundles, and in single myoblasts.

### Mutant desmin alters the axial myofibrillar lattice arrangement in fast- and slow-twitch muscle fibers of young R349P desmin knock-in mice

**Figure 2:**
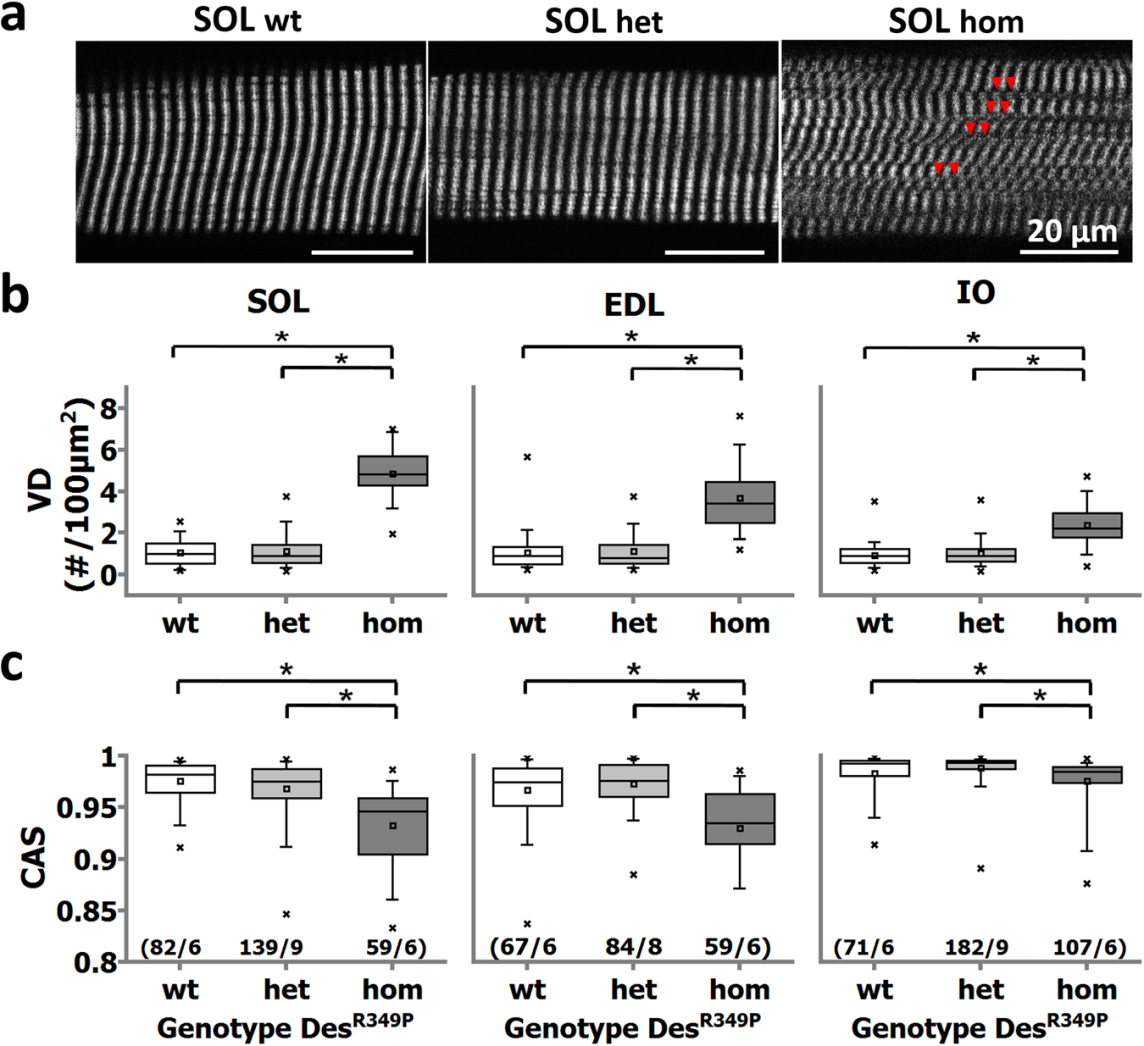
Morphometric analyses of cytoarchitecture in single muscle fibers from young Des^R349P^ mice using SHG microscopy. (**a**) Representative SHG images from the middle section of a single fiber from a wt (Des^wt/wt^), het (Des^R349P/wt^) and hom (Des^R349P/R349P^) knock-in mouse. Hom muscle fibers show prominent disruptions of the myofibrillar lattice. This is analyzed in detail in (**b**) for the number of *verniers* (*VD*), and in (**c**) for the *cosine angle sums* (CAS). VD and CAS were significantly increased and decreased, respectively, in single fibers from different muscles examined (SOL, EDL, IO) in hom mice. Het mice showed values similar to the wt. (n/m) denotes n fibers from m animals. *p < 0.05, one-way ANOVA with post-hoc Bonferroni-correction. Scale bar: 20 µm.

Figure 2a shows representative SHG images of a single SOL fiber of each genotype. The images clearly indicate an increase in myofibrillar axial lattice disruption from the het to the hom genotype. This is confirmed in a large number of single fibers from three different muscles (SOL, EDL, IO, details are given in the Methods) (Fig. 2b,c). While fibers from hom Des^R349P^ mice displayed significantly different VD and CAS values compared to wt mice, fibers from het mice showed only minor morphological alterations. These data demonstrate that both the axial lattice organization and the parallel myofibrillar orientation are compromised by the expression of mutant desmin in hom Des^R349P^ mice.

### Mutant desmin alters nuclear morphology and density in *soleus* muscle from young Des^R349P^ knock-in mice

**Figure 3:**
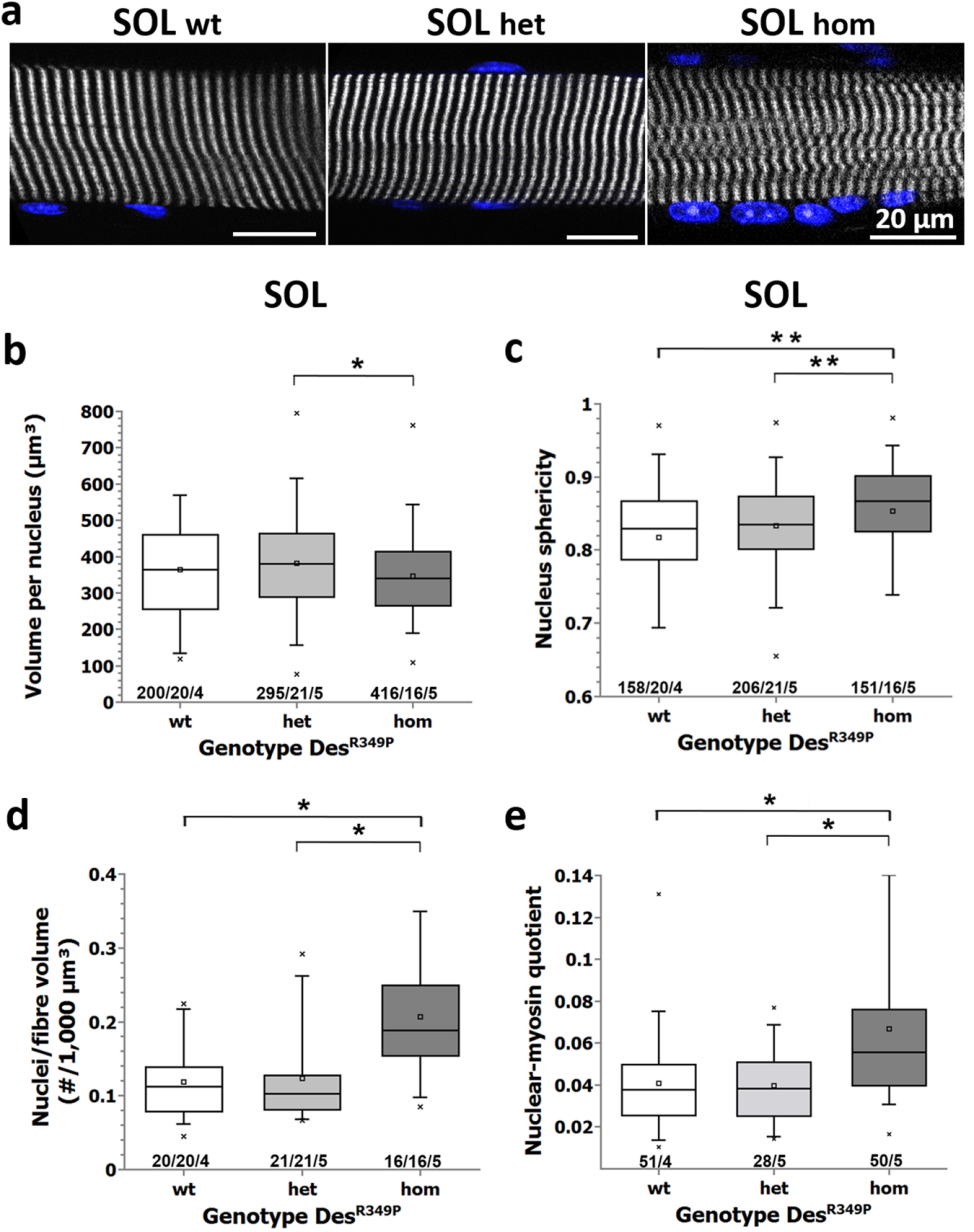
Morphometric analyses of nuclei point towards nuclear pathology in *soleus* fibers from young Des^R349P^ mice. (**a**) example images from the middle section of a single SOL fiber from a wt, het, and hom Des^R349P^ knock-in mouse showing the SHG (grey) and counterstained nuclear signal (blue). Although the volume per nucleus in fibers from hom animals was not different to wt (**b**), other morphological parameters were significantly different, e.g. hom fibers showing an increased rounded shape (sphericity) (**c**), or vastly increased number of nuclei per fiber volume (**d**), the latter two also compared to het Des^R349P^ mutations. The nuclear-to-myosin volume ratios were also largely increased in the hom Des^R349P^ genotype (**e**). (c/n/m) depicts (c) cell nuclei in (n) fibers from (m) mice. *p < 0.05, **p < 0.01, one-way ANOVA with Bonferroni-correction. Scale bar: 20 µm.

The desmin IF network is important for the positioning and anchorage of myonuclei^25^. In desmin- and DNA-double labeled isolated muscle fibers from hom Des^R349P^ mice, we noted a markedly rounder nuclear shape compared to wt fibers. To study this in depth, nuclei were simultaneously imaged in addition to SHG signals. Figure 3a shows example images from single SOL fibers of each genotype. In contrast to the normal localization and shape of nuclei at the periphery of wt and het fibers, nuclei in hom fibers were more often centrally located, had a decreased nuclear volume (Fig. 3b), displayed a significantly increased sphericity (Fig. 3c), and showed a significantly increased density (Fig. 3d). The latter was also reflected in increased nuclear-myosin ratios, pointing towards a markedly reduced biomotoric efficiency in the Des^R349P^ background, in particular in hom animals (Fig. 3e). Beyond a more central position of myonuclei - a typical morphological sign in a wide variety of myopathies - these data demonstrate that the expression of mutant desmin also inflicts a nuclear pathology.

### Mutant desmin is still targeted to Z-discs in *soleus* single muscle fibers

**Figure 4:**
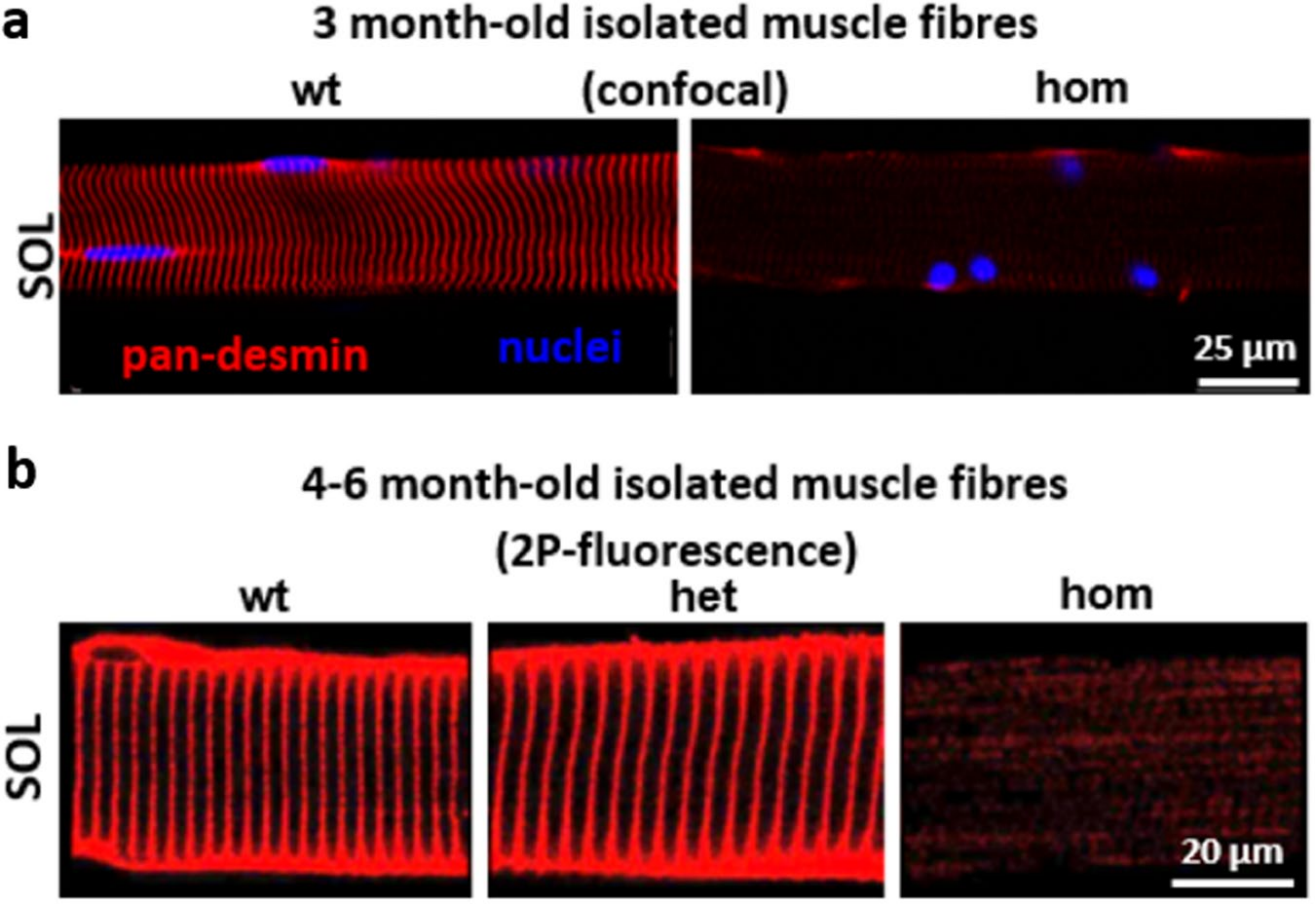
Desmin subcellular localization patterns in single fibers from *soleus* muscle of young Des^R349P^ mice. (**a**) Confocal images taken from single SOL muscle fibers from 3-month-old wt and hom Des^R349P^ mice. Note the vast reduction of regular cross-striated pattern and rounding of myonuclei in the hom fiber. (**b**) 2P-desmin-fluorescence in single muscle fibers from 5-month-old wt, het, and hom Des^R349P^ mice. Note that these latter desmin signals originate from a focal excitation volume about 1 µm in thickness, which corresponds to the scale of a single myofibril. While no apparent differences could be detected between wt and het fibers, the signal intensity in hom fibers was markedly reduced. However, the mutant desmin is still detectable in a cross-striated pattern indicating that its binding to the periphery of myofibrillar z-discs is still, at least in part, preserved.

Confocal image analysis of isolated SOL muscle fibers from hom mice demonstrated a nearly complete absence of the typical cross-striated desmin staining pattern as well as the presence of small subsarcolemmal protein aggregates (Fig. 4a). To analyze the specific subcellular localization of the mutant desmin within individual muscle fibers in more detail, we used 2-photon (2P)-fluorescence microscopy in single fibers. Using an antibody that detects both the wt and mutant desmin, 2P images show, as expected, the typical cross-striated pattern (wt, het). However, mutant desmin in hom SOL fibers also shows a weak but regular cross-striated staining pattern (Fig. 4b). This strongly indicates that mutant desmin, although unable to polymerize into a functional three-dimensional IF network^26^, retains its binding capacity to the periphery of myofibrillar Z-discs.

### Active biomechanics: altered contractile Ca^2+^ sensitivity and SR Ca^2+^ release induced force in *soleus* fiber bundles from young Des^R349P^ knock-in mice

**Figure 5:**
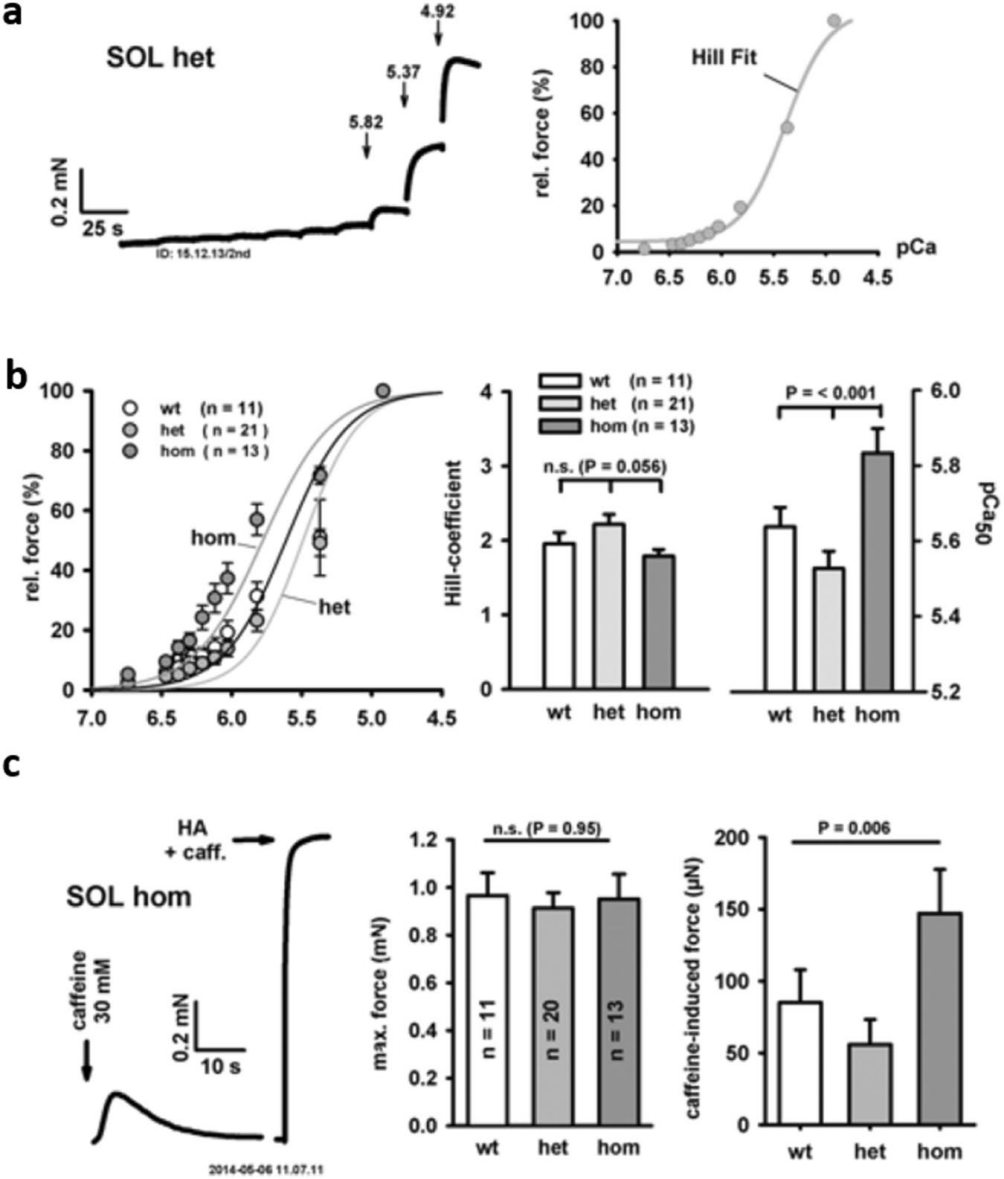
Active force biomechanics in small fiber bundles from *soleus* muscles of young Des^R349P^ mice. (**a**) Representative example recordings of force in a small SOL fiber bundle from a young het Des^R349P^ knock-in mouse with increasing Ca^2+^ concentrations (decreasing pCa) in the bath. The right panel shows the analysis of steady-state force at a given pCa along with the sigmoidal Hill fit to the data. (**b**) Group data from a number of bundles from wt and het and hom Des^R349P^ mice showing a substantial shift in het mice towards lower Ca^2+^-sensitivity, but a shift towards higher Ca^2+^-sensitivity in the hom mice, also reflected by a significantly larger pCa_50_ value in the latter. (**c**) Example recording from a small SOL fiber bundle from a young hom Des^R349P^ knock-in mouse showing a caffeine-induced force transient followed by maximum force at Ca^2+^-saturated condition (high activating Ca^2+^ and caffeine solution). Maximum absolute forces at saturating Ca^2+^ concentrations are similar in all three genotypes (middle panel) but caffeine-induced force transients are much larger in the hom over het and wt mice (right panel).

To investigate how the altered myofibrillar cytoarchitecture may impact on active muscle force production, we first determined the Ca^2+^ sensitivity of the contractile apparatus of small skinned SOL fiber bundles. Figure 5a shows a representative recording of force responses in different pCa environments with the typical staircase pattern with increasing Ca^2+^ concentrations (i.e. decreasing pCa values). Also shown are the force plateaus and the resulting sigmoidal Hill-fit of the force-Ca^2+^ relation (Fig. 5a). Figure 5b shows corresponding group data for each genotype. Notably, force-Ca^2+^ curves were shifted towards smaller pCa values for heterozygous and towards higher pCa values for homozygous Des^R349P^ bundles as compared to the wt. This is consistent with a reduced Ca^2+^ sensitivity (smaller pCa_50_ value) for het and an increased Ca^2+^ sensitivity (larger pCa_50_) for hom Des^R349P^ fibers. Additionally, hom bundles displayed a highly significant increase in pCa_50_ values when compared to the other two genotypes (Fig. 5b).

We next assessed caffeine-induced sarcoplasmic (SR) Ca^2+^-release-mediated force transients and maximum force levels at Ca^2+^ saturating conditions (HA + caff.) Fig. 5c). Maximum force was in the same range in all three genotypes however, force amplitudes of caffeine-induced force transients were significantly increased in the hom compared to het and wt genotypes (Fig. 5c). Thus, the increased sarcoplasmic Ca^2+^ release-induced force in hom SOL fiber bundles can be attributed to a higher Ca^2+^ sensitivity of the contractile apparatus in this genotype. These results provide insight into a mechanism that may balance (or even over-compensate, as shown here) compromised force production at physiological activation levels (i.e. submaximal Ca^2+^ activation) inflicted by the above described myofibrillar disarray in the early disease stages in homozygous mice.

### Passive biomechanics: increased axial stiffness and stretch-induced vulnerability in Des^R349P^*soleus* muscle fiber bundles

**Figure 6:**
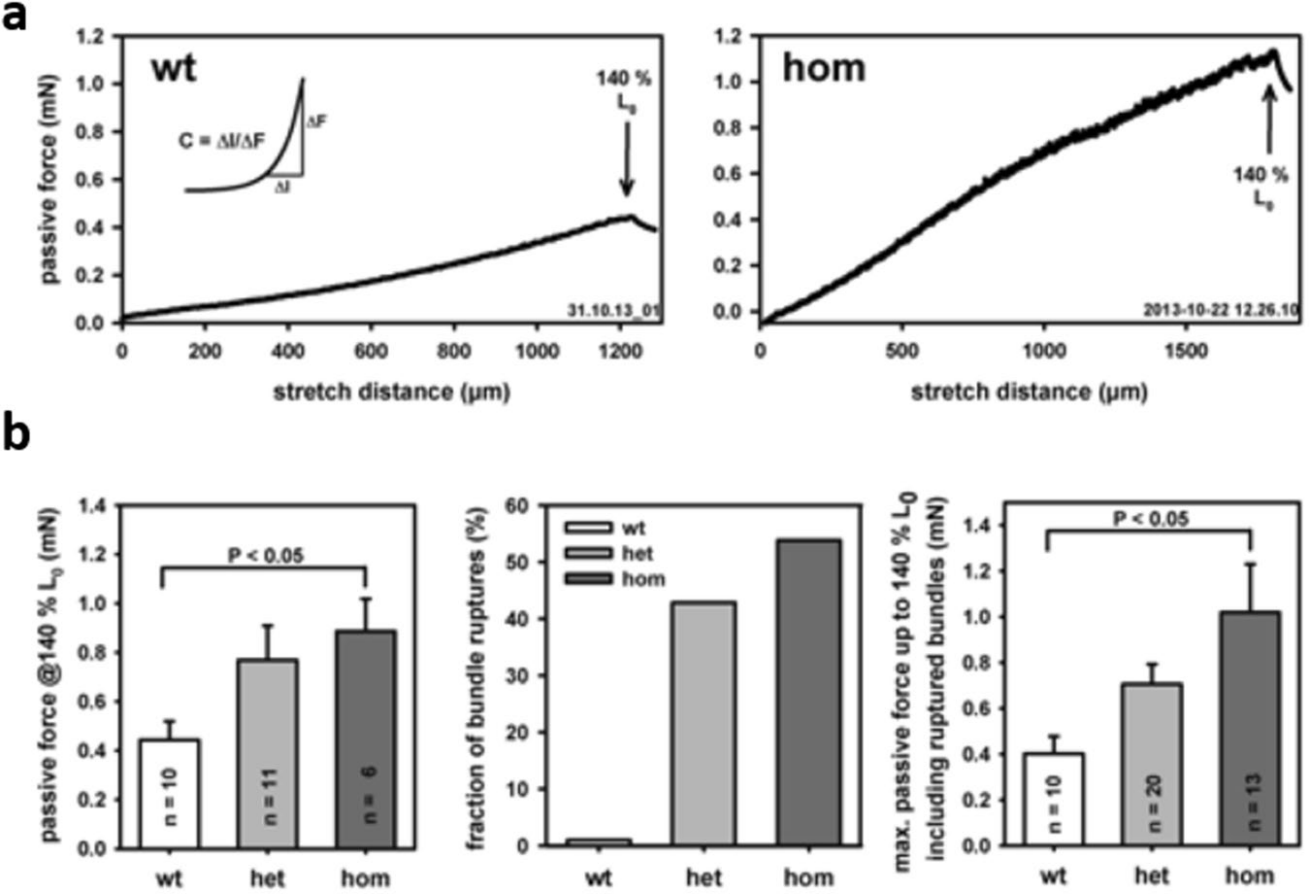
Passive resting length-tension curves in fiber bundles from young Des^R349P^ mice reveal reduced axial compliance. (**a**) Representative resting length-tension curves from slow quasi-static elongation experiments in a SOL bundle from a young wt and hom Des^R349P^ mouse. Bundles were stretched from L_0_ to 140% L_0_ and restoration force was recorded continuously. Some fiber bundles already ruptured before reaching the 140% L_0_ mark, in particular those with a Des^R349P^ genetic background. (**b**) Passive restoration force at 140% L_0_ is markedly increased in bundles from Des^R349P^ mice (significant for hom, left panel). A large fraction of Des^R349P^ carrying bundles ruptured before reaching the 140% L_0_ mark (middle panel) and when merging all maximum passive restoration forces from intact bundles at 140% L_0_ and bundles that ruptured before 140% L_0_ (force just before rupture point), the restoration force is even more increased in hom Des^R349P^ bundles, arguing for an even more diminished axial compliance in hom < het < wt bundles.

Next, we hypothesized that R349P mutant desmin may also negatively impact on passive biomechanics. To assess the global axial elasticity of small fiber bundles, resting length-tension curves were recorded. Figure 6a shows resting length-tension curve recordings from a wt and a hom Des^R349P^ SOL fiber bundle. Notably, the hom bundle displayed markedly larger restoration forces compared to the wt at equivalent stretch. This effect was statistically significant for all hom bundles. Somewhat larger passive restoration forces were also seen in het fiber bundles (Fig. 6b). Moreover, bundles from both Des^R349P^ genotypes were strikingly more fragile than bundles from wt animals, with nearly 50% of mutant bundles rupturing before reaching 140% L_0_ extensions (Fig. 6b). When those ruptured bundles were included in the analysis of the maximum restoration force during stretch before rupture or after reaching 140% L_0_, the restoration force of hom bundles was even further increased compared to wt and het bundles. This indicates a markedly diminished axial compliance (i.e. higher axial elastic stiffness) in the homozygous background that also explains higher fragility upon stretch. Notably, there was also a trend for an increased passive axial stiffness in het vs. wt bundles (Fig. 6b).

### Mutant desmin-inflicted changes of visco-elastic behavior: increased stiffness of sarcolemmal components and altered viscosity of sarcomeric elements

**Figure 7:**
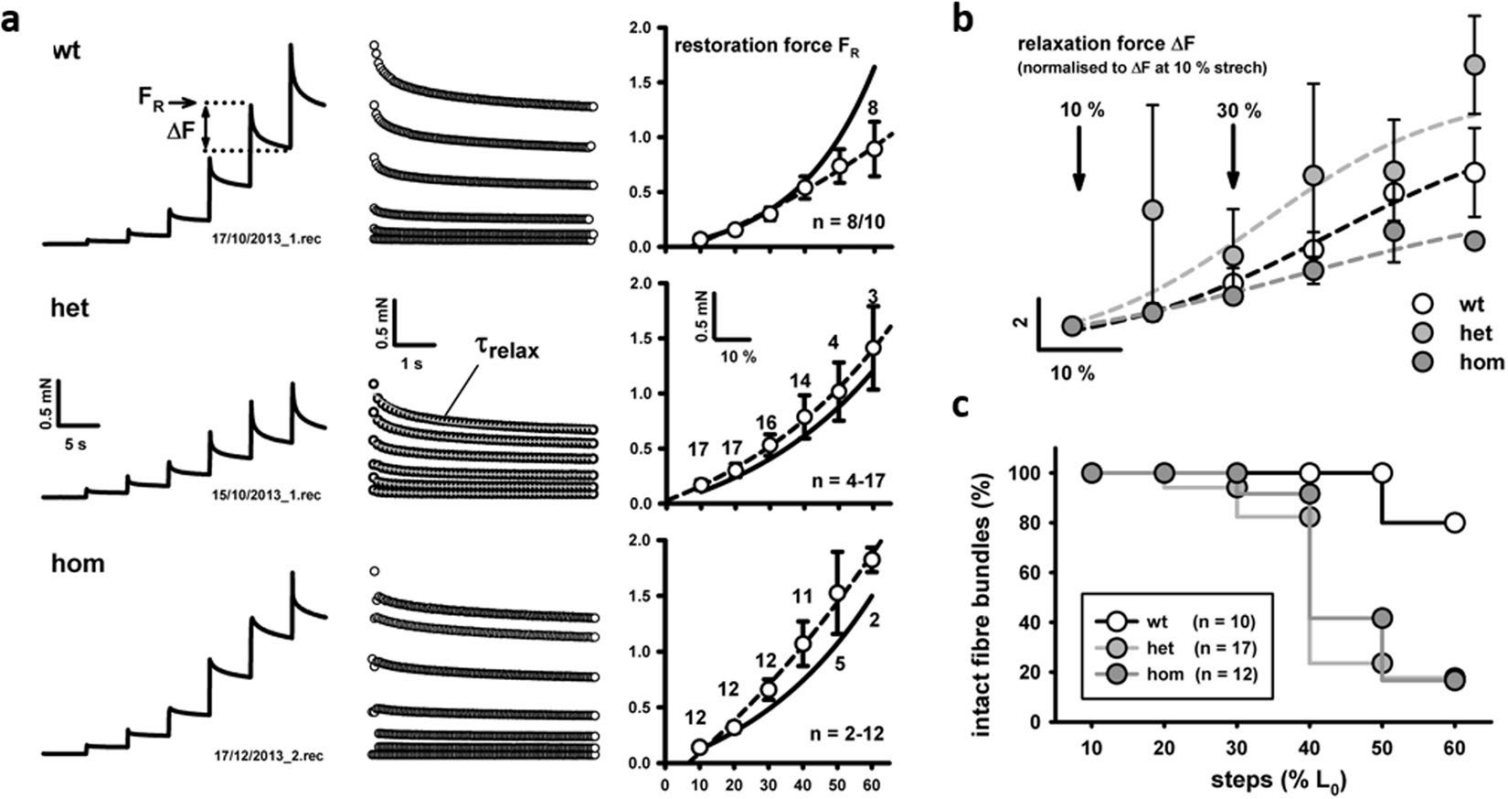
Visco-elastic properties of small fiber bundles from *soleus* muscles of young Des^R349P^ knock-in mice. (**a**) Representative example recordings of passive force during fast length-extension jumps in 10% of L_0_ intervals. Fiber bundles were acutely stretched and kept at that length for 5 s before proceeding to the next 10% stretch (range: 10–60% L_0_). Force responses at each step consist of an instantaneous restoration force (F_R_) followed by a relaxation phase during which force declined to a plateau. The F_R_ values, time constants during exponential force relaxation (τ_relax_) and ΔF during relaxation were analyzed. F_R_-length relations are much steeper in the Des^R349P^ mutated preparations over the wt and are steeper for the hom over the het mutation (right panel in (**a**): solid line refers to mean exponential curve reconstructed from the mean parameters of each individual curve fit; dashed line refers to exponential fit through the mean data). (**b**) ΔF-ΔL relationships showing the mean relaxation data for each genotype. Those indicate a more reduced relaxation in the hom genotype than in the wt. (**c**) Maier-Kaplan curves showing the percentage of still intact bundles at a given stretch jump.

To additionally determine the visco-elastic behavior of SOL fiber bundles from Des^R349P^ mice, ‘stretch-jump’ experiments were performed (sudden stretches at 10% L_0_ intervals followed by 5 s holding phase). As shown in Fig. 7a, bundles responded to each stretch step with an instantaneous increase in restoration force F_R_, followed by an exponential relaxation with a time constant τ_relax_ to a plateau force. These experiments reflect the kinetics of viscous relaxation of the sarcomeric (e.g. titin), extra-sarcomeric (e.g. IF-network), and membranous components (e.g. caveolins) in response to sudden stretch. The relaxation kinetics was not different among genotypes (Fig. 7a). Instead, the F_R_-stretch relationships (Fig. 7a) were much steeper in the Des^R349P^ background, especially in the homozygous knock-in compared to the wt. However, group data for ΔF values suggest a compromised viscoelastic behavior only in the hom but not in the het genotype (Fig. 7b). Nonetheless, both hom and het Des^R349P^ SOL bundles were much more prone to stretch-induced rupture, as shown by the Kaplan-Maier curves (Fig. 7c). Although the viscous relaxation force amplitude ΔF of het bundles is only slightly larger than in wt bundles, this might not sufficiently counter-balance their increased F_R_ and may therefore, explain a similar susceptibility to stretch-induced rupture as in hom bundles (Fig. 6b). Taken together, the dynamic passive biomechanics data in Des^R349P^ SOL bundles also document an increased dynamic stiffness and changes in the viscous relaxation of passive elements, which together with the increased static axial stiffness reported above explains their predisposition to stretch-induced damage.

### Axial stiffness of isolated myofibrillar *soleus* bundles in Des^R349P^ mice

**Figure 8:**
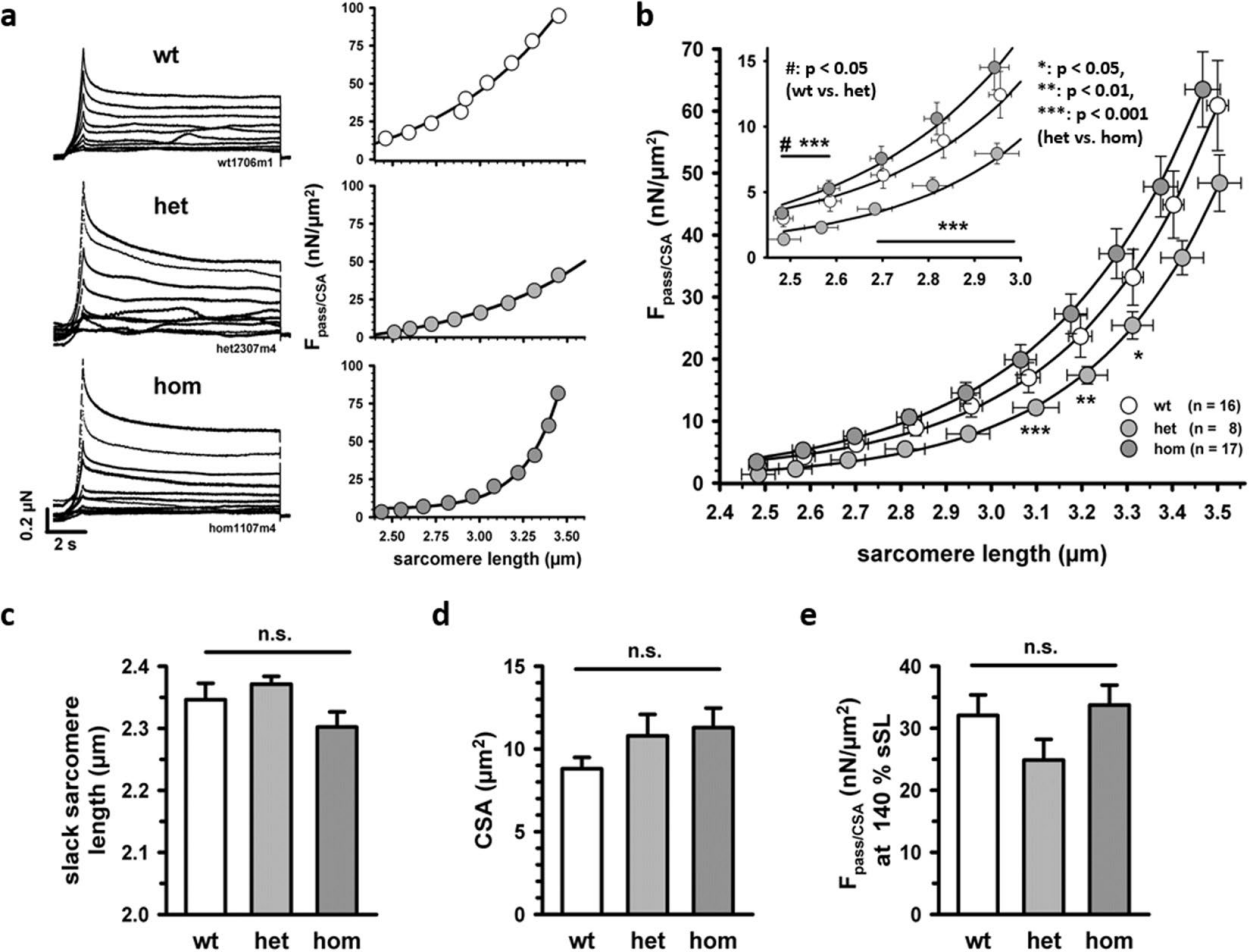
Passive mechanical properties of myofibrillar bundles from *soleus* muscles of young Des^R349P^ knock-in mice. (**a**) Exemplary traces of myofibrillar bundles stretched under relaxing conditions (pCa 8) at constant speed for 1 s from 8 to 180% of L_0_. (**b**) Mean data ± SEM of actual sarcomere lengths and passive tension, i.e. passive force normalized to the cross-sectional area of the myofibrillar bundle (F_pass_/CSA), from 16 bundles of wt, 8 bundles of het and 17 bundles of hom Des^R349P^ mice. The averaged group data for each genotype is fitted to a worm-like chain model of entropic elasticity (solid lines). No significant difference between bundles from hom and wt mice was found while bundles from het mice had significantly lower passive tension at moderate stretch compared to bundles from hom mice. *p < 0.05, **p < 0.01, and ***p < 0.01, indicated by Student’s t-test. At low stretch amplitude, bundles from het mice exhibited slightly lower tension than wt bundles (^#^ *p* < 0.05). (**c**) Slack sarcomere length, (**d**) cross-sectional area, and (**e**) passive tension at 140% L_0_ of the myofibrillar bundles were not different among the three different genotypes (ANOVA).

We next determined the passive steady-state tension-sarcomere length and elasticity behavior of sarcomeric elements in subcellular myofibrillar bundles. The few-µm thin myofibrillar bundles prepared from detergent-treated SOL muscle do no longer contain the membranous components of axial visco-elasticity seen in myofiber bundles. Thus, this preparation allows to specifically probe the stiffness of the sarcomeres in conjunction to the desmin network interlinking the myofibrils at the extra-sarcomeric Z-discs. Figure 8a shows superimposed traces of increasing stretch (individual traces from 8% to 80% of L_0_) in a representative wt, het, and hom myofibrillar bundle (left panels) alongside with the steady-state passive tension-sarcomere length relationship (right panels). The individual recordings suggest a steeper curve for hom myofibrillar bundles, an observation that was confirmed in several bundles. For het myofibrillar bundles, the steady-state tension-sarcomere length relationship is shifted downwards to lower tensions. Group data (Fig. 8b) show a somewhat larger passive tension of hom myofibrillar bundles at a given sarcomere length as compared to the wt, although this was not statistically significant. Het myofibrillar bundles, however, showed a lower passive force as compared to both wt and hom bundles, which was highly significant, in particular for lower extensions. Thus, passive tension of myofibrillar bundles was oppositely affected in het and hom mice, similarly as was observed for the Ca^2+^-sensitivity of force generation in the fiber bundles. Slack sarcomere lengths (Fig. 8c), cross-sectional area (Fig. 8d), and passive tension at a particular myofibrillar length of 140% of L_0_ (Fig. 8e) were not significantly different among the three genotypes. This indicates that in contrast to the multicellular preparation, which still includes the effect of membranous and some extracellular matrix components, the axial stiffness of sarcomeres probed in the myofibrillar bundles is not substantially increased in hom Des^R349P^ myofibrillar bundles (reflecting the subcellular level). Moreover, the axial stiffness even seems decreased in the heterozygous myofibrillar bundles, corroborating that the increased restoring forces observed in multicellular preparations may result from predominant stiffening of probably membrane-associated cytoskeletal components.

### Lateral stiffness of the membrane complex in Des^R349P^ myoblasts: increased in heterozygous but unaltered in homozygous cells

**Figure 9:**
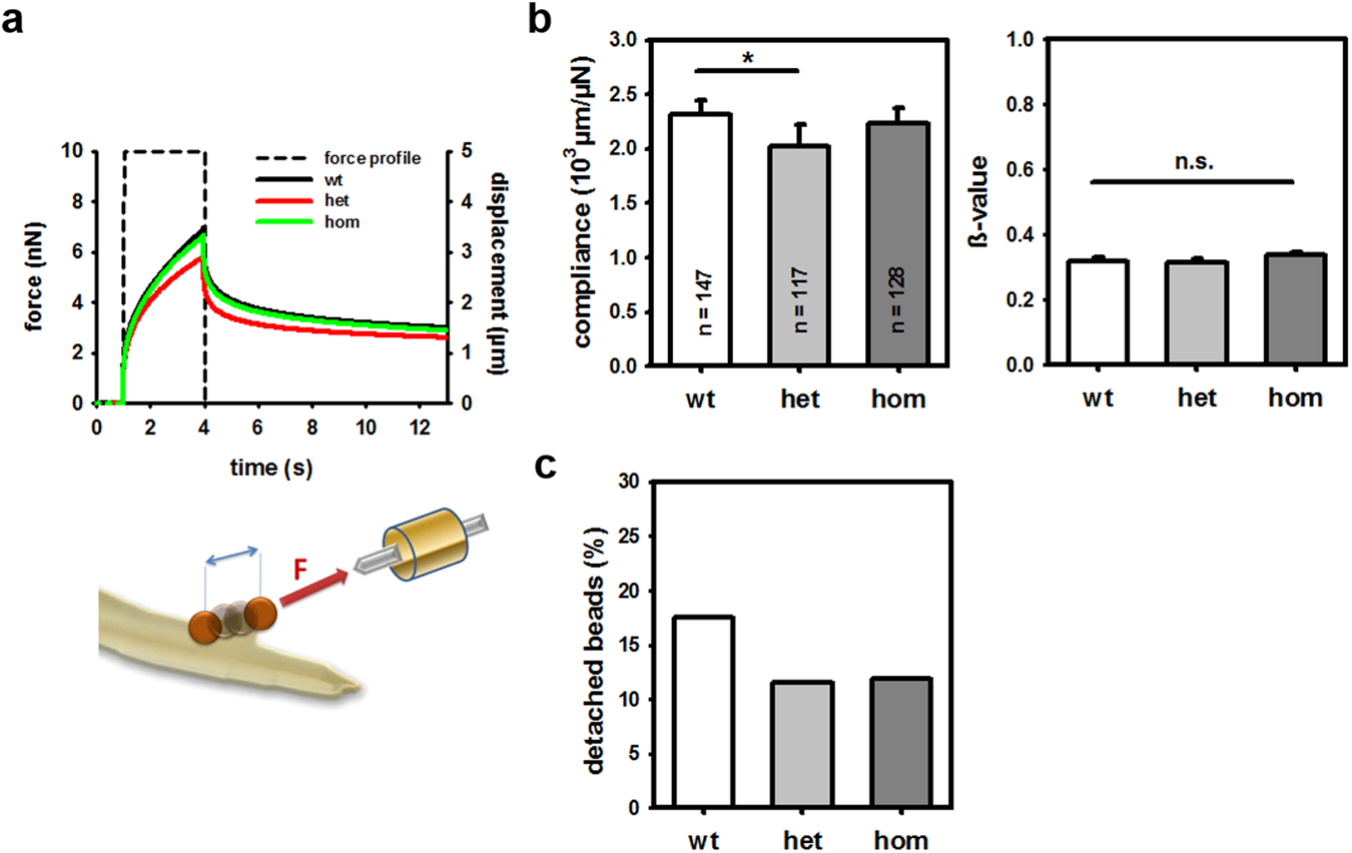
Lateral passive biomechanics properties from myoblasts derived from wt, het, and hom Des^R349P^ mice. (**a**) Schematics of the magnetic tweezer experiments applying a constant magnetically-induced force of 10 nN for 3 s to myoblasts into which magnetic fibronectin-coated micro-beads had been coated to the membrane focal adhesion complexes. Shown are mean displacement curves during the ‘force on’ and ‘force off’ phase from which the stiffness and visco-elastic behavior were extracted. (**b**) Lateral compliance values (median values due to log-normal distribution) of the membrane and near-membrane cytoskeleton complex in 147 wt, 117 het, and 128 hom myoblasts as well as the visco-elastic parameter ß (mean values). (**c**) Percentage of detached beads during the ‘force-on’ phase.

The desmin cytoskeleton provides a crucial mechanical link from the Z-disc of the myofibrillar apparatus via costameres to the extra-sarcomeric cytoskeleton. Thus, it also contributes importantly to the lateral biomechanical properties of muscle cells, e.g. lateral stiffness. To measure lateral stiffness directly, we performed magnetic tweezer micro-rheology in immortalized R349P desmin-knock-in muscle cell cultures. We applied forces of 10 nN for 3 s to fibronectin-coated superparamagnetic beads attached to integrin receptors on the surface of myoblasts (Fig. 9a). The bead displacement (d) after a step increase of the force (F) followed a power law with time (t) as described by ref. 27. The cell’s lateral compliance *J*_0_, which is inversely proportional to its stiffness, was determined from the creep response *J*(*t*) of the cells by fitting the displacement with the typical power law response $J(t)=\frac{{J}_{0}}{F}\cdot {(\frac{t}{{t}_{0}})}^{\beta }$, where ${t}_{0}=1s$. Only beads which remained attached to the cell during the whole force application were evaluated. The lateral compliance *J*_0_ was significantly decreased in het R349P desmin myoblasts as compared to the wt and hom genotype (Fig. 9b). The value *β*, which is a measure of the cells’ visco-elastic behavior, remained similar for all cell lines (Fig. 9b). Moreover, the decreased number of detached beads of het and hom R349P desmin myoblasts compared to wt reflects an increased binding strength between fibronectin-coated beads and cytoskeleton-linked adhesion complexes (Fig. 9c). Thus, these data demonstrate that het Des^R349P^ myoblasts are less compliant and are therefore, less deformable than hom and wt cells.

## Discussion

We used a high-resolution microscopy and multilevel biomechanical approach to analyze early disease stages of autosomal-dominant and recessive desminopathies in muscle fiber and myofibrillar bundles, and myoblasts from Des^R349P^ knock-in mice. As new findings of our study, we describe vastly altered myofibrillar and nuclear cytoarchitecture, increased axial elastic steady-state stiffness (from resting-length tension curves) and differentially altered Ca^2+^-sensitivity of the contractile apparatus (increased in mice carrying two mutated alleles, decreased in heterozygous animals) at early stages of R349P desminopathy.

### Altered cytoarchitecture revealed by multiphoton quantitative morphometry in early desminopathy

Our SHG microscopy analyses of young R349P desmin knock-in mice demonstrated marked myofibrillar lattice disruptions (seen in *vernier* densities) and angular disorder of myofibrillar orientation (quantified as *cosine angle sums*) in various muscles from hom mice. Though addressed by classical light and electron microscopic approaches, similar disruptions of the myofibrillar organization have previously been detected, but not quantitated in the context of human desminopathies and desmin knockout mice^5, 28–30^. Notably, slow-twitch SOL muscles were more severely affected compared to fast-twitch muscles, like IO and EDL. This is in line with increased disease susceptibility of slow-twitch muscles^21^. However, in R349P desmin knock-in mice, we did not observe fiber branching and splitting, which was reported for *flexor digitorum brevis* muscle of 8–12 month old desmin knockout mice^31^. Our combined SHG/2-photon microscopy approach further shows that the homozygous expression of mutant desmin impacts on the number/density, shape, and volume of nuclei in skeletal muscle fibers. Morphological evidence of nuclear pathology (central nuclei) in desminopathies has been described before^19, 21, 28^. However, the functional consequences on nuclear signaling, transcription, nuclear pore size, and substrate exchange are still unknown. The increased number of nuclei per volume and nuclear shape changes may point towards an increased demand for nuclear/transcriptional activity^32–34^, reflected by a deduced lower ‘biomotoric efficiency’ in hom fibers, which may be a mechanism to compensate for a faster breakdown of muscle proteins. An increase in the number of nuclei would fit into the previously reported finding that mutant desmin induces an increased turnover of desmin protein species and other key proteins of the extra-sarcomeric cytoskeleton^21^.

The myofibrillar lattice disruptions and nuclear pathology in hom R349P desmin knock-in mice, and the lack of these changes in het mice, need to be discussed in relation to the known faulty assembly properties of the R349P desmin mutant. Transfection and *in vitro* assembly studies demonstrated that R349P/R350P mutation aborts the normal desmin filament assembly process at an early stage^26^. As a consequence, the mutant desmin in hom mice is not capable to form a functional *de novo* desmin network^26^, and the mutant protein is sequestered into sarcoplasmic protein aggregates^21^. Thus, the inability of the mutant desmin to build a functional filament network is likely to account for the striking lattice disruptions and aberrant orientation of the myofibrillar apparatus as well as the changes in nuclear morphology. Since het mice express a mixture of wt and mutant R349P desmin, the situation is far more complex. Using various mixtures of recombinant wt and mutant desmin, we demonstrated in a previous study that the presence of 25% of the mutant desmin effectively aborted the normal polymerization process^26^. Given the multi-nuclear nature of skeletal muscle fibers, disruptions of the desmin network in heterozygous patients and mice are likely to occur only in subcellular areas, in which the amount of mutant desmin may exceed the critical threshold of 25%. Focal enrichment of mutant desmin may interfere with the binding of wt desmin to its myofibrillar interaction partners and lead to disturbances of the desmin network formed by the wt protein.

### Increased axial elastic stiffness and increased myofibrillar Ca^2+^-sensitivity in early homozygous desminopathy

Elasticity is a key biophysical property of desmin intermediate filaments. An analysis of the tensile properties of single desmin filaments demonstrated that they can be mechanically stretched up to 3.4-fold of their initial lengths and become more visco-elastic in response to mechanical extension^6^. These nonlinear tensile properties of desmin IFs are thought to exert a pivotal role in storing and dissipating mechanical energy during muscle contraction. Our biomechanical analysis of early disease stages in het and hom R349P mice revealed identical levels of maximum force production in small fiber bundles under Ca^2+^ saturating conditions as in wt littermates. However, while het mice displayed a reduced contractile Ca^2+^-sensitivity, hom animals unexpectedly exhibited an increased Ca^2+^-sensitivity. Notably, SOL fiber bundles from hom mice, which showed marked changes in the myofibrillar cytoarchitecture as seen in SHG images, also generated increased force amplitudes in caffeine-induced force transients. The latter finding, which can likely be attributed to this higher Ca^2+^-sensitivity of the contractile apparatus, points towards a mechanism that even over-compensates the projected decline in active force production due to the altered myofibrillar architecture.

The Ca^2+^-sensitivity of skeletal muscle is generally thought to be dependent on the phosphorylation status of myofibrillar proteins^35, 36^, as well as the relative fraction of slow- and fast-type myosin heavy chain (MHC) isoforms. Since normal SOL muscle mostly contains slow-twitch MHC isoform type I (MHC I) and fast-twitch MHC type IIA isoforms^37^, it is possible that the genotype-dependent changes in myofibrillar Ca^2+^-sensitivity may be related to a switch in the overall fiber type composition in this particular muscle or, alternatively, the phosphorylation status of the myosin light chains^38–40^. Notably, we found a shift in MHC I proportions in homogenates from *soleus* muscle towards a higher percentage in homozygous Des^R349P^ mice (~65%) over wt (~45%) and heterozygous (~40%) mice in preliminary SDS PAGE analyses (data not shown). This provides one possible explanation for the higher Ca^2+^-sensitivity and thus, higher force levels in hom Des^R349P^ soleus muscle in our study, since other studies that used simultaneous assessment of myofibrillar Ca^2+^-sensitivity and MHC isoform fiber typing established a higher Ca^2+^-sensitivity in rodent MHC I fibers over MHC II fibers^38^. The genotype-specific changes of Ca^2+^-sensitivity seen in fibers, i.e. the increased sensitivity of hom compared to wt and decreased sensitivity of het compared to wt fibers, are mirrored by similar genotype-specific changes in passive myofibrillar tension, which was lowest for het and highest for hom bundles. This coincidence corroborates the idea that desminopathies cause adaptational mechanisms that alter the biomechanical properties of the sarcomeres, in particular in the relaxed and submaximally activated muscle. Our findings do not rule out additional impact on myosin light chain phosphorylation status, an issue that will be addressed in future studies.

Other key findings of our study were an increase in the overall axial stiffness and an enhanced stretch-induced vulnerability. Although this overall finding has been already concluded from our previous study^21^, that earlier study only used fast stretch ‘jump’ protocols to assess visco-elastic behavior in a few fiber bundles to determine increased susceptibility to stretch-induced axial rupture in hom bundles at 140% L_0_ stretch amplitudes. The present study corroborates on this in a larger number of bundles but also provides new insights into steady-state compliance being vastly reduced in hom bundles as seen in larger steepness of the steady-state resting length-tension curves (Fig. 6). Though axial stiffness was more pronounced in hom fibers, it is noteworthy that a large portion of fiber bundles from both hom and het mice ruptured before they reached 140% stretch relative to the resting length L_0_. An increased stiffness was also seen in stretch jump experiments on fiber bundles as well as myofibrillar preparations. In the latter, however, the effect was less prominent, with a tendency towards larger stiffness of hom myofibrils. Notably, increased stiffness of SOL muscle fibers was also reported in desmin knockout mice^41^. These data demonstrate that any derangement of the extra-sarcomeric desmin cytoskeleton results in an increased axial muscle stiffness to render muscle more vulnerable to mechanical stress.

### Increased lateral sarcolemmal stiffness in undifferentiated mutant R349P desmin carrying myoblasts

Since the desmin cytoskeleton also provides lateral myofibrillar stability^3^, we studied the impact of mutant desmin on the lateral stiffness in immortalized R349P desmin knock-in muscle cells via magnetic tweezer microrheology. These studies demonstrated an increased stiffness in heterozygous myoblasts, indicating a reduced deformability compared to immortalized wt myoblasts. The latter findings mirror previous results in cultured primary myoblasts from a patient with a het R350P desmin mutation^27^. The analysis of hom immortalized R349P desmin-knock-in myoblasts, however, revealed lateral compliance levels that were in the same range as in wt cells. Thus, increased lateral sarcolemmal stiffness in undifferentiated myoblasts seems to be restricted to a genetic situation in which mutant R349P desmin is co-expressed with wt desmin. Further studies are required to resolve the molecular mechanisms of differential affection of axial vs. lateral stiffness in R349P carrying muscle preparations.

### Clinical relevance and limitations of the mouse model

Both het and hom knock-in animals develop dilated cardiomyopathy, conduction defects, and cardiac arrhythmias. However, significant skeletal muscle weakness was only seen in hom mice aged 19 months or older but not in het animals^21^. This is in contrast to the clinical picture in human desminopathies, where progressive muscle weakness usually develops in the second to the forth decade of life for dominant, and in first to the second decades for recessive mutations^12^. When reflecting the overall clinical and myopathological similarities as well as discrepancies between our het and hom R349P desmin knock-in models and human desminopathy patients, homozygous R349P desmin knock-in mice, which serve as a model for autosomal-recessive desminopathies, share more commonalities with the human pathology than their heterozygous littermates. However, since het R349P desmin knock-in mice express nearly the same ratio of mutant to wt desmin protein as human heterozygous R350P desminopathy patients^21^, the lack of skeletal muscle weakness in het mice points towards additional factors needed for developing a muscle pathology. With regard to the latter issue, it is noteworthy that heterozygous W2711X filamin C knock-in mice, a patient-mimicking mouse model for filamin C-related myofibrillar myopathy, showed a markedly increased extent of myofiber pathology in response to acute high-intensity exercise^42^. As a result, the late onset or lack of skeletal muscle weakness in R349P desmin knock-in mice may primarily be related to the limited physical activity of these laboratory animals kept in a sedentary condition. Since human tissue at preclinical stages is usually not available, in this study, we focused on muscle preparations from young het and hom Des^R349P^ mice to explore early pathophysiological aspects of altered structure-function relationships that could explain the developing muscle weakness preceding the clinical weakness in desminopathies.

### Summary and outlook

In summary, our high-resolution microscopy and biomechanical study in R349P desmin knock-in mice unveils a multiplicity of novel, mutant desmin-induced cellular effects in early stages of autosomal-dominant and -recessive desminopathies^10, 13^. The primary pathophysiological denominator is the mutant desmin-induced pathology of the extra-sarcomeric cytoskeleton, which subsequently (i) impacts on axial and lateral orientation of myofibrils, (ii) number and morphology of myonuclei, (iii) axial stiffness of muscle fibers, predominately in slow-twitch muscle, and (iv) vulnerability of muscle fibers in response to mechanical stress. Our new data strongly argues against the hypothesis that these widespread alterations are simply the consequence of the presence of sarcoplasmic protein aggregates. The present delineation of morphological and biomechanical parameters defining the early disease changes is an important basis for future studies addressing the influence of physical exercise as well as pharmacological interventions.

## Methods

### R349P desmin knock-in (DesR349P) mice

To study early disease stages, we used 17–23 weeks old adult heterozygous (hereafter termed het) and homozygous (hereafter termed hom) R349P desmin knock-in mice (B6J.129Sv-Destm1.1Ccrs; synonym, B6J.129Sv-Destm1(R349P)Cscl&Rfsr^21^. All animal related work was performed in accordance with the German Animal Welfare Act (Tierschutzgesetz) as well as the German Regulation for the protection of animals used for experimental purposes or other scientific purposes (Tierschutz-Versuchstierordnung). Investigations were approved by the governmental Office for Animal Care and Use (Regierung von Mittelfranken, Ansbach, Germany; ref. TS-14/2015). Details can be found the Supporting Information (SI).

### Generation of Des^R349P^ immortalized skeletal muscle cell cultures

Myoblasts were isolated according to a modified protocol^43, 44^. Details are given in the SI.

### Muscle fiber, small fiber bundle, myofibrillar bundle and myocyte preparations

After inhalation anesthesia with isoflurane, mice were killed by cervical dislocation and the hind limbs were cut off. The *soleus* muscle (SOL), the *extensor digitorum longus* muscle (EDL) and the *interossei* toe muscles (IO) were dissected under a stereo-microscope (SMZ 745T, Nikon). For multiphoton microscopy and morphometry studies, relaxed muscles were fixed in TBS with 1% (v/v) non-acidic formaldehyde solution (Carl Roth GmbH, Karlsruhe, Germany) for at least 72 h at 4 °C. Single fibers from SOL and EDL were obtained through manual tethering. IO single fibers were obtained by enzymatic digestion.

For multicellular biomechanics experiments, small fiber bundles of five single fibers were dissected from the unfixed SOL muscles.

For myofibrillar bundle preparations, SOL muscles from 19 ± 1 week old mice (wt, het, hom) were used.

For single myocyte biomechanics recordings, immortalized (p53-deficient) mouse myoblasts homozygous and heterozygous for the Des^R349P^ mutation and controls carrying the wt desmin were used. Myoblasts were cultured in growth medium.

### Nuclear staining protocols and desmin immunofluorescence

Subcellular distribution of desmin in single fibers was visualized by immunofluorescence using a primary desmin antibody (Clone D33, M0760, Dako, Hamburg, Germany). Details are given in the SI.

### *Second Harmonic Generation* (SHG) and multiphoton fluorescence (MPF) imaging

Single fibers (SOL, EDL, IO) were imaged using an ultra-fast high-performance multifocal multiphoton microscope (TriMScope II, LaVision BioTec, Bielefeld, Germany). A mode-locked ps-pulsed Ti:Sa laser (Chameleon Vision II, Coherent, Santa Clara, CA, USA) tuned to 800 nm was used to simultaneously excite the *Second Harmonic Generation* (SHG) signal of myosin and the respective fluorescent signals. Details are given in the SI.

### Image processing and morphometric analysis of SHG and MPF data

An automated image processing algorithm written in MATLAB (MathWorks, Natick, MA, USA), based on a boundary tensor was used for morphometric analysis of 3D SHG images. Details and parameters of the algorithm are given in refs 45, 46 and 47 and in the SI.

### SDS PAGE analysis of myosin heavy chain distributions in *soleus* muscle homogenates

MHC I distribution over MHC II was obtained by SDS PAGE separation of myosin heavy chain isoforms from *soleus* muscle homogenates. Details are given in the SI. MHC I and MHC II (IIa, IIb not further distinguished) were set to 100% and MHC I signal densities expressed as fraction of MHC I + II.

### Assessment of active and passive biomechanics in small fiber bundles

SOL fiber bundles were subjected to active and passive force recordings involving caffeine-induced Ca^2+^-release-mediated force transients, pCa-force recordings, steady-state resting length-tension curves and quick stretch-force response curves. For details, refer to the SI.

### Myofibrillar bundle biomechanics

Force measurements in isolated myofibrillar bundles from SOL muscles were performed in relaxing solution^48, 49^. Details are given in the SI.

### Magnetic tweezer compliance recordings in single myoblasts

For compliance recordings of immortalized wt, het, and hom R349P cultured myoblasts, a magnetic tweezer device was used as described previously^27^. Details are given in the SI.

### Statistical analysis

For single fiber morphometry data, one-way ANOVA (Sigma Plot, Systat Software, Erkrath) was applied on the three genotypes (wt, het and hom), followed by post-hoc analysis with Bonferroni correction. p < 0.05 was considered significant (*) and p < 0.01 highly significant (**). Normality of data was tested using the Shapiro–Wilk test. Data are presented as box plots (median value: line, quartiles: whiskers 5–95 percentiles, minimum/maximum values: x, mean: rectangle). For biomechanics data, one-way ANOVA tests were applied followed by Bonferroni correction or Student’s two-sided unpaired t-test. Biomechanics data are presented as mean ± SEM with number of observations, n.

## Electronic supplementary material

Supplementary Information

## References

[1] Wang K, Ramirez-Mitchell R (1983). A network of transverse and longitudinal intermediate filaments is associated with sarcomeres of adult vertebrate skeletal muscle. The Journal of cell biology.

[2] Reipert S (1999). Association of mitochondria with plectin and desmin intermediate filaments in striated muscle. Experimental cell research.

[3] Schroder R (1999). Immunogold EM reveals a close association of plectin and the desmin cytoskeleton in human skeletal muscle. European journal of cell biology.

[4] Capetanaki Y, Bloch RJ, Kouloumenta A, Mavroidis M, Psarras S (2007). Muscle intermediate filaments and their links to membranes and membranous organelles. Experimental cell research.

[5] Shah SB (2004). Structural and functional roles of desmin in mouse skeletal muscle during passive deformation. Biophysical journal.

[6] Kreplak L, Herrmann H, Aebi U (2008). Tensile properties of single desmin intermediate filaments. Biophysical journal.

[7] Goldfarb LG (1998). Missense mutations in desmin associated with familial cardiac and skeletal myopathy. Nature genetics.

[8] Dalakas MC (2000). Desmin myopathy, a skeletal myopathy with cardiomyopathy caused by mutations in the desmin gene. The New England journal of medicine.

[9] Schroder R, Vrabie A, Goebel HH (2007). Primary desminopathies. Journal of cellular and molecular medicine.

[10] Schroder R, Schoser B (2009). Myofibrillar myopathies: a clinical and myopathological guide. Brain Pathol.

[11] Schroder R (2013). Protein aggregate myopathies: the many faces of an expanding disease group. Acta neuropathologica.

[12] van Spaendonck-Zwarts KY (2012). Recurrent and founder mutations in the Netherlands: the cardiac phenotype of DES founder mutations p.S13F and p.N342D. Netherlands heart journal: monthly journal of the Netherlands Society of Cardiology and the Netherlands Heart Foundation.

[13] Clemen CS, Herrmann H, Strelkov SV, Schroder R (2013). Desminopathies: pathology and mechanisms. Acta neuropathologica.

[14] Arbustini E (1998). Restrictive cardiomyopathy, atrioventricular block and mild to subclinical myopathy in patients with desmin-immunoreactive material deposits. Journal of the American College of Cardiology.

[15] Cetin N (2013). A novel desmin mutation leading to autosomal recessive limb-girdle muscular dystrophy: distinct histopathological outcomes compared with desminopathies. Journal of medical genetics.

[16] Munoz-Marmol AM (1998). A dysfunctional desmin mutation in a patient with severe generalized myopathy. Proceedings of the National Academy of Sciences of the United States of America.

[17] Pinol-Ripoll G (2009). Severe infantile-onset cardiomyopathy associated with a homozygous deletion in desmin. Neuromuscular disorders: NMD.

[18] Carmignac, V., Sharma, S., Arbogast, S., Fischer, D., Serreri, C., Serria, M., Stoltenburg, G., Maurage, C. A., Herrmann, H., Cuisset, J. M., Bär, H. & Ferreiro, A. A homozygous desmin deletion causes an Emery-Dreifuss like recessive myopathy with desmin depletion. *Neuromusc*. *Disord*. **19** (2009).

[19] Henderson M (2013). Recessive desmin-null muscular dystrophy with central nuclei and mitochondrial abnormalities. Acta neuropathologica.

[20] McLaughlin HM (2013). Compound heterozygosity of predicted loss-of-function DES variants in a family with recessive desminopathy. BMC Med Genet.

[21] Clemen CS (2015). The toxic effect of R350P mutant desmin in striated muscle of man and mouse. Acta neuropathologica.

[22] Plotnikov SV, Millard AC, Campagnola PJ, Mohler WA (2006). Characterization of the myosin-based source for second-harmonic generation from muscle sarcomeres. Biophysical journal.

[23] Both M (2004). Second harmonic imaging of intrinsic signals in muscle fibers *in situ*. Journal of biomedical optics.

[24] Friedrich O (2010). Microarchitecture is severely compromised but motor protein function is preserved in dystrophic mdx skeletal muscle. Biophysical journal.

[25] Chapman MA (2014). Disruption of both nesprin 1 and desmin results in nuclear anchorage defects and fibrosis in skeletal muscle. Human molecular genetics.

[26] Bar H (2005). Pathogenic effects of a novel heterozygous R350P desmin mutation on the assembly of desmin intermediate filaments *in vivo* and *in vitro*. Human molecular genetics.

[27] Bonakdar N (2012). Biomechanical characterization of a desminopathy in primary human myoblasts. Biochemical and biophysical research communications.

[28] Olive M (2004). Desmin-related myopathy: clinical, electrophysiological, radiological, neuropathological and genetic studies. Journal of the neurological sciences.

[29] Fichna JP (2014). Two desmin gene mutations associated with myofibrillar myopathies in Polish families. PloS one.

[30] Schroder R (2003). On noxious desmin: functional effects of a novel heterozygous desmin insertion mutation on the extrasarcomeric desmin cytoskeleton and mitochondria. Human molecular genetics.

[31] Goodall MH, Ward CW, Pratt SJ, Bloch RJ, Lovering RM (2012). Structural and functional evaluation of branched myofibers lacking intermediate filaments. American journal of physiology. Cell physiology.

[32] Nava MM, Fedele R, Raimondi MT (2016). Computational prediction of strain-dependent diffusion of transcription factors through the cell nucleus. Biomechanics and modeling in mechanobiology.

[33] Thomas CH, Collier JH, Sfeir CS, Healy KE (2002). Engineering gene expression and protein synthesis by modulation of nuclear shape. Proceedings of the National Academy of Sciences of the United States of America.

[34] Dahl KN, Ribeiro AJ, Lammerding J (2008). Nuclear shape, mechanics, and mechanotransduction. Circulation research.

[35] Stepkowski D, Osinska H, Szczesna D, Wrotek M, Kakol I (1985). Decoration of actin filaments with skeletal muscle heavy meromyosin containing either phosphorylated or dephosphorylated regulatory light chains. Biochimica et biophysica acta.

[36] Szczesna D (2002). Phosphorylation of the regulatory light chains of myosin affects Ca2+ sensitivity of skeletal muscle contraction. J Appl Physiol (1985).

[37] Friedrich O, Weber C, von Wegner F, Chamberlain JS, Fink RH (2008). Unloaded speed of shortening in voltage-clamped intact skeletal muscle fibers from wt, mdx, and transgenic minidystrophin mice using a novel high-speed acquisition system. Biophysical journal.

[38] Bortolotto SK, Stephenson DG, Stephenson GM (1999). Fiber type populations and Ca2+-activation properties of single fibers in soleus muscles from SHR and WKY rats. The American journal of physiology.

[39] Bowslaugh J, Gittings W, Vandenboom R (2016). Myosin light chain phosphorylation is required for peak power output of mouse fast skeletal muscle *in vitro*. Pflugers Archiv: European journal of physiology.

[40] Greenberg MJ (2009). The molecular effects of skeletal muscle myosin regulatory light chain phosphorylation. American journal of physiology. Regulatory, integrative and comparative physiology.

[41] Anderson J, Li Z, Goubel F (2001). Passive stiffness is increased in soleus muscle of desmin knockout mouse. Muscle & nerve.

[42] Chevessier F (2015). Myofibrillar instability exacerbated by acute exercise in filaminopathy. Human molecular genetics.

[43] Rosenblatt JD, Lunt AI, Parry DJ, Partridge TA (1995). Culturing satellite cells from living single muscle fiber explants. In vitro cellular & developmental biology. Animal.

[44] Winter L (2014). Chemical chaperone ameliorates pathological protein aggregation in plectin-deficient muscle. The Journal of clinical investigation.

[45] Buttgereit A, Weber C, Garbe CS, Friedrich O (2013). From chaos to split-ups–SHG microscopy reveals a specific remodelling mechanism in ageing dystrophic muscle. The Journal of pathology.

[46] Garbe CS, Buttgereit A, Schurmann S, Friedrich O (2012). Automated multiscale morphometry of muscle disease from second harmonic generation microscopy using tensor-based image processing. IEEE transactions on bio-medical engineering.

[47] Buttgereit A, Weber C, Friedrich O (2014). A novel quantitative morphometry approach to assess regeneration in dystrophic skeletal muscle. Neuromuscular disorders: NMD.

[48] Stehle R, Kruger M, Pfitzer G (2002). Force kinetics and individual sarcomere dynamics in cardiac myofibrils after rapid ca(2+) changes. Biophysical journal.

[49] Stehle R (2002). Isometric force kinetics upon rapid activation and relaxation of mouse, guinea pig and human heart muscle studied on the subcellular myofibrillar level. Basic research in cardiology.

