# Supplementary material for: Early signs of architectural and biomechanical failure in isolated myofibers and immortalized myoblasts from desmin-mutant knock-in mice
Source: Sci Rep. 2017 May 3;7:1391. doi: 10.1038/s41598-017-01485-x (PMC5431221; doi:10.1038/s41598-017-01485-x)
Supplement: Supplementary file 1 — Supplementary Information [file 41598_2017_1485_MOESM1_ESM.pdf]

Supplemental Information

Early signs of architectural and biomechanical failure in isolated myofibers and immortalized myoblasts from desmin-mutant knock-in mice

Stefanie Diermeier[1,2,8], Julian Iberl[1], Kristina Vetter[1], Michael Haug[1,8], Charlotte Pollmann[1,8], Barbara Reischl[1,8], Andreas Buttgereit[1,8], Sebastian Schürmann[1,2,8], Marina Spörrer[3,8], Wolfgang H. Goldmann[3,8], Ben Fabry[3,8], Fatiha Elhamine[4], Robert Stehle[4], Gabriele Pfitzer[4], Lilli Winter[5], Christoph S. Clemen[6,7], Harald Herrmann[5,8], Rolf Schröder[5,8,*,#], Oliver Friedrich[1,2,8,*,#]

1 Institute of Medical Biotechnology, Friedrich-Alexander Universität Erlangen-Nürnberg, Paul-Gordan-Str.3, 91052 Erlangen
2 SAOT, Erlangen Graduate School in Advanced Optical Technologies, Friedrich-Alexander-Universität Erlangen-Nürnberg
3 Department of Physics, Biophysics Group, Friedrich-Alexander-Universität Erlangen-Nürnberg, Erlangen, Germany
4 Institute for Vegetative Physiology, University of Cologne, Germany
5 Institute of Neuropathology, University Hospital Erlangen, Germany
6 Institute of Biochemistry I, Medical Faculty, University of Cologne, Germany
7 Department of Neurology, Heimer Institute for Muscle Research, University Hospital Bergmannsheil, Ruhr-University Bochum, Bochum, Germany
8 Muscle Research Center Erlangen (MURCE), Friedrich-Alexander-Universität Erlangen-Nürnberg

*: corresponding author e-mail: oliver.friedrich@mbt.uni-erlangen.de
(for technological expertise, Lead Contact)
rolf.schroeder@uk-erlangen.de
(for medical expertise)

#: equally contributing senior authors

R349P desmin knock-in mice

In this disease model, in which the expression of the mutant desmin is controlled by the endogenous gene regulation sites, the het and hom mice develop phenotypes that correspond to the autosomal-dominant and -recessive human desminopathies, respectively. As controls, wild-type (hereafter termed wt) littermates were used. All animal-related work was performed in accordance with the German Animal Welfare Act (Tierschutzgesetz) as well as the German Regulation for the protection of animals used for experimental purposes or other scientific purposes (Tierschutz-Versuchstierverordnung). The investigations were approved by the governmental Office for Animal Care and Use (Regierung von Mittelfranken, 91511 Ansbach, Germany; reference number TS-14/2015). All applicable international, national, and institutional guidelines for the care and use of animals were followed. Mice were 17 - 23 weeks of age.

Small fiber bundles, single muscle fiber, and myofibrillar bundle preparations

After inhalation anesthesia with isoflurane, mice were killed by cervical dislocation. The tail tip was stored at -20 °C for genotyping. The hind limbs were cut off and immersed in Ringer solution (Ri; in mM: NaCl, 145, Hepes, 10, glucose, 10, KCl, 5, $CaCl_2$, 2.5, $MgCl_2$, 1, pH 7.4), and the *soleus* muscle (SOL), the *extensor digitorum longus* muscle (EDL) and the *interossei* toe muscles (IO) were dissected under a stereo-microscope (SMZ 745T, Nikon). SOL and EDL were pinned under slight stretch to elastomer-coated culture dishes (Sylgard 184, Dow Corning), and solution was exchanged to a ‘high K^+‘-relaxing solution (HKS, in mM: K-glutamate, 140, Hepes, 10, glucose, 10, $MgCl_2$, 10, EGTA, 1, pH 7.0) for 30 min at 4 °C. For multiphoton microscopy and morphometry

studies, the relaxed muscles were chemically fixed in TBS with 1 % (v/v) non-acidic formaldehyde solution (Carl Roth GmbH, Karlsruhe, Germany) for at least 72 h at 4 °C. Single fibers from SOL and EDL were obtained through manual tethering using fine forceps. Small pieces of sticky tape were used to attach each single fiber to grease strips in the recording chamber filled with TBS prior to multiphoton imaging. The fixation procedure increased the stability of the muscle fibers and preserved their ultrastructure for SHG imaging. The preparation of fixed single fibers showed markedly less preparation-induced and time-dependent structural artifacts due to chemical decomposition by autolysis than native single fiber preparations.

IO single fibers were obtained by enzymatic digestion. The muscles were incubated in Ri supplemented with 0.5 mg/ml collagenase (type IA; Sigma-Aldrich, Taufkirchen, Germany) for 45 min at 37 °C. The enzymatic digestion was stopped by washing with Ri at RT, and the IO fibers were gently isolated by trituration using fire-polished Pasteur pipettes. IO single fibers were transferred to a recording chamber, and 1 % formaldehyde was added to the muscle suspension.

For multicellular biomechanics experiments, small fiber bundles of five single fibers were dissected from the unfixed SOL muscles in HKS solution. We restricted our biomechanical experiments to the SOL muscle as it showed the most prominent morphological pathology in R349P desmin knock-in mice [21]. Fiber bundles were mounted between a force transducer pin (KG7, Scientific Instruments, Heidelberg, Germany) and a software-controlled voice coil actuator (SMAC CAL12-010-51BSA, Ispringen, Germany). The bundle preparation was then lowered into a rack of serially arranged wells containing different sets of internal solutions for relaxing the bundles or activating SR Ca^{2+}-release-induced force (see below). The dwell time and well identifiers

were controlled with LabView. An automated set of biomechanical recordings on the same preparation was then started, consisting of sequential runs of (i) caffeine-induced Ca^{2+}-release dependent force transients, (ii) pCa-force curves, (iii) passive resting length – tension curves, and (iv) fast stretch steps (details see below).

For myofibrillar bundle preparations, SOL muscles from 19 $\pm$ 1 week old mice (wt, het, hom) were used. To dissolve all membranous structures, muscles were covered with an ice-cold solution containing 0.5 % Triton-X100, 5 mM K-phosphate, 5 mM potassium azide, 2 mM Mg-acetate, 5 mM K_2EGTA, 3 mM Na_2ATP and 47 mM potassium creatine phosphate (pH 7.0), cut into thin strips and incubated at 0 °C. After 1 h, the solution was replaced by an identical solution without Triton and stored for up to 48 h at 0 °C. Immediately before the experiment, the muscle strips were homogenized at 0 °C for 5 s at maximum speed with a blender (Ultra-Turrax T25, Janke & Kunkel, Staufen, Germany).

For single myocyte biomechanics recordings, immortalized (p53-deficient) mouse myoblasts homozygous and heterozygous for the Des^{R349P} mutation and controls carrying the wt desmin were used. Myoblasts were cultured in growth medium.

Nuclear staining protocols and desmin immunofluorescence

Fixed single fibres (SOL, EDL, IO) were incubated in Ringer's solution (Ri) supplemented with 10 µM Hoechst 33342 nucleic acid stain (PK-CA707-40047, PromoCell) for 2 h at RT with a final washing procedure. The subcellular distribution of desmin in the fixed single fibres was visualized by immunofluorescence using a primary desmin antibody (Clone D33, M0760, Dako, Hamburg, Germany). To reduce background signals, the fibers were treated three times with 0.1 M glycine (3908.2, Carl

Roth GmbH + Co KG, Karlsruhe, Germany) in TBS for 5 min. After washing in TBS for 10 min, the fibers were permeabilized using 0.25 % Tween 20-TBS (9127.1, Carl Roth) solution for 20 min. Blocking was performed with 5 % bovine serum albumin (BSA A7030, Sigma-Aldrich) in TBS for 1 h at RT. The desmin antibody was incubated 1:200 in 5 % BSA-TBS shaking overnight at 4 °C. After washing, the secondary antibody Alexa Fluor 594 F(ab`)$_2$ fragment (A11020, Molecular Probes Life Technologies) was added at 1:10,000 in 5 % BSA-TBS for 1 h followed by a three times 10 min washing step.

Second Harmonic Generation (SHG) and multiphoton fluorescence (MPF) imaging

The recorded images had a size of 150 x 150 µm and consisted of 1072 x 1072 pixels. Two scans of each pixel at 600 Hz were averaged to increase the signal-to-noise ratio. The average laser power at the sample was around 16 mW, and pulse duration was around 2 ps, with a repetition rate of approximately 80 MHz. A symmetric transmitted light configuration of two water immersion objectives was used for detection. On the excitation side (backscattered, descanned), an LD C-Apochromat lens (40x/1.1/UV-VIS-IR/WD 0.62, Carl Zeiss, Jena, Germany), and on the transmission side (forward scattered, non-descanned), a W Plan-Apochromat lens (20x/1.0/(UV)VIS-IR/WD 1.88/DIC M27 75mm, Carl Zeiss) were used. A 377/50 nm BrightLine single band pass filter (FF01-377/50-25, Semrock Inc., Rochester, New York, USA) was inserted to block the incident laser beam. The SHG signal was detected by an ultrasensitive, non-descanned transmission photo multiplier tube (PMT) (H 7422-40 LV 5M, Hamamatsu Photonics). Single fibers were z-scanned using a 0.5 µm step size at a software magnification of ×3 to detect *verniers* (voxel-size: 0.139 × 0.139 × 0.500 µm^3) and to derive cosine angle sums. Multiphoton excited fluorescence (MPF) of the nuclei and

desmin were detected as backward scattered signals simultaneously to the forward scattered SHG signal at 400 nm. The nuclear signal at 460 nm and the desmin signal at 620 nm were separated using an ultra-flat laser dichroic mirror with single band wavelength range from 450 nm to 597 nm (ZT594RDC, 229225, Chroma Technology group, Acal BFi Germany GmbH, Gröbenzell).

Generation of R349P desmin knock-in immortalized skeletal muscle cell cultures

S*oleus* (SOL) muscles from an 8-week-old mouse were enzymatically dissociated in 6 ml enzyme solution (0.2 % collagenase I, (Gibco), in DMEM) for 1.5 – 2 h at 37° C with gentle agitation. The digested tissue was poured into a 55 mm cell culture dish containing 6 ml pre-warmed DMEM, and single muscle fibers were released by gentle trituration with a Pasteur pipette. Cell culture dishes and glass pipettes were pre-flushed with 10 % horse serum in DMEM. Once 20 - 30 intact fibers were separated, the muscle bulk was transferred to a fresh dish. The previous dish, containing separated fibers and debris, was stored in the incubator at 37° C and 5 % CO_2. The fiber separation cycle was repeated until sufficient numbers of fibers had been dissociated from the muscle. The intact muscle fibers were then separated from the debris by transferring them in a Pasteur pipette to a dish coated with Matrigel (BD Biosciences; diluted 1:100 in DMEM). The plated fibers were allowed to settle and attach for 3 min to the Matrigel substrate, and then 1 ml plating medium (DMEM containing 10 % horse serum and 0.5 % chick embryo extract) was slowly added to each dish. Plates were returned to the incubator at 37° C and 5 % CO_2 for 24 h. Myoblasts were split, pre-plated on uncoated culture dishes for up to 2 h (to remove contaminating fibroblasts, performed at every splitting procedure), and finally cultivated in Ham's F10 medium supplemented with 20 % FCS,

2.5 ng/ml basic fibroblast growth factor (bFGF, Promega), and 1 % penicillin/streptomycin on collagen-coated (0.01 % collagen in PBS) culture dishes. Immortalized myoblast cell lines with passage numbers of up to 40 were used for experiments.

Image processing and morphometric analysis of SHG and MPF data

Y-shaped deviations from the sarcomere pattern in a z-stack of SHG images were defined as *verniers* [23]. The density of *verniers* (VD) [46] was weighted according to the single fiber area per slice (a_i) and was presented as weighted number of *verniers* within a total area (A) of 100 µm^2. The normalized VD was given by:

$$\overline{VD} = \frac{\sum_i a_i \, VD_i}{\sum_i a_i} = \frac{\sum_i a_i \, VD_i}{A}$$

The *cosine angle sum* (CAS) [24] was used as a direct measure for the coherency and structural integrity of single fibers, i.e. reflecting the degree of local angular deviation of myofibrillar bundles from the main trunk axis [45]. The CAS was calculated as weighted mean according to the fiber area of all slices of a z-stack (Ω) by using $|\Omega|$ as number of pixels representing the surface of the fiber, Φ (*x*,*y*) as local direction, and the median [Φ (*x*,*y*)] as the main direction of the fiber:

$$CAS = \frac{1}{|\Omega|} \sum_{(x,y) \epsilon \Omega} \cos\{\phi\,(x,y) - median\,[\phi(x,y)]\}$$

The two- and three-channel (SHG, Hoechst and Alexa Fluor 594) z-stacks were displayed with Fiji based on ImageJ (National Institutes of Health, Bethesda, MD, USA). The segmentation operation by thresholding extracted the nucleus volume (NV) and myosin volume (MV) by counting the segmented voxels (nHoechst, nSHG). Using these parameters, the nuclear-myosin-quotient (NMQ) was calculated as:

$$NMQ = \frac{NV}{MV} = \frac{nHoechst}{nSHG}$$

The corresponding biomotoric efficiency (BE) was derived from NMQ as described before as NMV [47]. The nuclear density and the 3D morphology of the nuclei was analyzed using IMARIS software (Bitplane AG, Zurich, Switzerland). The number of nuclei per fiber volume (nuclear density), the volume per nucleus (μm^3), and the nucleus sphericity in single fibers were calculated using the Surpass view – contour surface menu of IMARIS.

SDS PAGE analysis of myosin heavy chain distributions in soleus muscle homogenates.

Myosin heavy chain isoforms (MHC) in whole muscle homogenates of frozen SOL muscle samples were electrophoretically separated on 8 % acrylamide separation gels containing 32 % glycerol (3783.1, Carl Roth GmbH, Karlsruhe, Germany), 200 mM Tris (pH 8.8) 100 mM glycine, 0.4% SDS, 0.1% APS (9592.3, Carl Roth) and 0.05% TEMED (2367.1, Carl Roth). Stacking gels consisted of 32% glycerol, 6% AA, 70 mM Tris (pH 6.8), 4 mM EDTA, 0.4 % SDS, 0.1 % APS and 0.05 % TEMED. After loading 10 µg of SOL homogenate in 2-fold loading dye onto the gels, 1 mM DTT (6908.2, Carl Roth) was added to the inner running buffer and run at 90 V for 20 min and then at 165 V for around 6 h at 4 °C. After overnight staining at RT with Roti Blue staining solution, gels were imaged with the Fusion-FX7-Spectra system and analysed with the Fiji gel analyser tool. MHC isoforms were analysed as in [37].

Assessment of active and passive biomechanics in small fiber bundles

Soleus fiber bundles were subjected to a series of active and passive force recordings. After attaching a bundle to the force transducer and voice coil actuator pin, the preparation was chemically permeabilized in high relaxing (HR) solution containing (mM): Hepes 30, $Mg(OH)_2$ 6.25, EGTA 30, Na_2ATP 8, Na_2CP (creatine phosphate) 10, pH 7.2, supplemented with 0.1 % (w/v) saponin for 90 s. HR was always used after steps where Ca^{2+} was released from the SR by means of caffeine (release solution, RS: low relaxing solution (LR, mM: Hepes 30, $Mg(OH)_2$ 7.86, $C_5H_8KNO_4$ 87.7, HDTA 6.6, EGTA 0.4, Na_2ATP 8, Na_2CP 10, pH 7.2) and 30 mM caffeine) or following maximum activation at a pCa of 4.92 in highly activating solution (HA, mM: Hepes 30, $Mg(OH)_2$ 6.05, EGTA 30, $CaCO_3$ 29, Na_2ATP 8, Na_2CP 10, pH 7.2). The SR was loaded in a controlled manner by incubation in an internal solution containing ~200 nM free Ca^{2+} for defined times. Ca^{2+}-sensitivity of the contractile apparatus (pCa-force) was assessed by immersing the preparation in various HR:HA mixtures with known pCa and recording force until steady-state levels were reached. Passive force properties comprised of quasi-static resting length-tension curves and fast 'stretch jumps'. For the former, the bundles were kept in LR solution at resting length L_0 (~2 mm), and the voice coil actuator was set to a constant translation speed away from the force transducer pin (velocity: 0.44 µm/s). Passive restoration force was continuously recorded, and resting length-tension curves were transformed from the time-force curves using the known extension velocity. The restoration force at 140 % L_0 was analyzed for each bundle as a measure for steady-state compliance at that extension. Passive visco-elastic bundle behaviour was assessed with fast 'stretch jump' protocols. Starting from L_0, bundles were immediately stretched in 10 % bins at very fast velocity and kept for 5 s at the new

length before proceeding to the next extension. The force response consisted of an instantaneous restoration force followed by an exponential relaxation to a steady-state force level F_{ss} at the given stretch bin. For analysis, the peak restoration force F_R, the difference between F_R and F_{ss}, ΔF, and the time constant of exponential relaxation τ_{relax} were determined in each bundle.

Myofibrillar bundle biomechanics

Force measurements in isolated myofibrillar bundles were performed in relaxing solution (pCa 8) containing 3 mM K_4Cl_2EGTA, 10 mM imidazole, 1 mM $K_2Cl_2Na_2MgATP$, 3 mM $MgCl_2$, 47.7 mM Na_2CrP, 2 mM DTT, pH 7.0 at 10 °C using the experimental setup described in [45,46]. Thin subcellular myofibrillar bundles (diameters of 3 – 4 µm) were mounted between the tip of an atomic force cantilever and the tip of a length-driving stiff tungsten needle. After mounting, the slack sarcomere length, the overall slack length L_0, and the diameter of the bundles were determined. To determine the relation of the passive steady-state tension versus the sarcomere length, the bundles were rapidly stretched by moving the tungsten needle with a piezo motor using variable length steps with sizes of 8 %-multiples of L_0. After stretch, an image of the bundle was taken using an ORCA-ER camera (Hamamatsu, Hamamatsu Photonics, Germany) and 60x objective (60x/0.70 Ph2 LCPlanFl, Olympus) for evaluating the actual sarcomere length using the software Aquacosmos (Hamamatsu Photonics). After holding the bundle for 12 s at stretched length, the bundle was rapidly slackened to determine its passive force from the drop of force to zero. Passive tension was calculated by normalizing passive force to the cross-sectional area and then plotted against the actual sarcomere length.

Magnetic tweezer compliance recordings in single myoblasts

For each experiment, 8 x 10^4 myoblasts were seeded overnight in a culture dish. 30 min before experiments, cells were incubated with fibronectin-coated paramagnetic beads of 4.5 µm diameter (Invitrogen). A magnetic field was generated using a solenoid with a needle-shaped core (HyMu80 alloy, Carpenter, Reading, PA). The needle tip was placed at a distance of 20 – 30 µm from a bead bound to the cell using a motorized micromanipulator (Injectman NI-2, Eppendorf). During measurements, bright-field images were taken by a CCD-camera (ORCAER, Hamamatsu) at a rate of 40 fps. The bead position was tracked using an intensity-weighted center-of-mass algorithm. Measurements on multiple beads per well were performed at 37 ^{o}C for 1 h, using a heated microscope stage on an inverted microscope at 40x magnification (NA 0.6) under bright-field illumination. The bead displacement (d) after a step increase of the force (F; 10 nN for 3 s to fibronectin-coated superparamagnetic beads attached to integrin receptors on the surface of myoblasts) followed a power law with time (t). The cell's lateral compliance J_0, which is inversely proportional to its stiffness, was determined from the creep response $J(t)$ of the cells by fitting the displacement with the typical power law response $J(t) = \frac{J_0}{F} \cdot \left(\frac{t}{t_0}\right)^{\beta}$, where $t_0 = 1s$.
